# Supplementary material for: Nurse-led telerehabilitation intervention to improve stroke efficacy: Protocol for a pilot randomized feasibility trial
Source: PLoS One. 2023 Jun 2;18(6):e0280973. doi: 10.1371/journal.pone.0280973 (PMC10237469; doi:10.1371/journal.pone.0280973)
Supplement: S1 Fig — (DOCX) [file pone.0280973.s001.docx]

**Fig 1. Enrolment, intervention and assessment schedule**

| **Study Period** | | | | | | | |
| --- | --- | --- | --- | --- | --- | --- | --- |
|  |  |  |  |  |  |  |  |
|  | **Screening** | **Allocation** | **Baseline** | **Intervention** | **Post intervention** | **Follow up 1** | **Follow up 2** |
| **Timepoint** | February 2023 to March,2023 | March, 2023 | 0 | 1 to 24 weeks |  | 4wks post intervention | 12 weeks post-intervention |
| **Enrollment** |  |  |  |  |  |  |  |
| Screening eligible participants | X |  |  |  |  |  |  |
| Informed consent | X |  |  |  |  |  |  |
| Randomization | X |  |  |  |  |  |  |
| **Allocation** |  | X |  |  |  |  |  |
| **Assessment** |  |  |  |  |  |  |  |
| Socio-demographic characteristics | X |  |  |  |  |  |  |
| History of condition | X |  |  |  |  |  |  |
| Cognitive function status |  |  | X | X | X |  |  |
| Functional status |  |  | X | X | X |  |  |
| Emotional status |  |  | X | X | X |  |  |
| Stroke self-efficacy |  |  | X |  | X | X | X |
| Satisfaction |  |  |  |  | X |  |  |
| Qualitative experiences |  |  |  |  | X |  |  |
| **Interventions** |  |  |  |  |  |  |  |
| Nurse-led intervention + Treatment as usual |  |  |  |  |  |  |  |
| Treatment as usual only |  |  |  |  |  |  |  |
