# Supplementary material for: Nurse-led telerehabilitation intervention to improve stroke efficacy: Protocol for a pilot randomized feasibility trial
Source: PLoS One. 2023 Jun 2;18(6):e0280973. doi: 10.1371/journal.pone.0280973 (PMC10237469; doi:10.1371/journal.pone.0280973)
Supplement: S2 Fig — (DOC) [file pone.0280973.s002.doc]

Fig 2. Workflow chart

**Allocation**

**Analysis**

**Follow-Up**

**Enrollment**

Assessed for eligibility (n= x)

Excluded (n= x)

♦  Not meeting inclusion criteria (n= x )

♦  Declined to participate (n= x )

♦  Other reasons (n= x)

Analysed (n= x)
♦ Excluded from analysis (give reasons) (n= x)

Lost to follow-up (give reasons) (n= x)

Discontinued intervention (give reasons) (n= x)
Immediately, 30days and 90 days after interventions

Allocate to intervention (n= 20)

♦ Received allocated intervention (n= x)

♦ Did not receive allocated intervention (give reasons) (n= x)

Lost to follow-up (give reasons) (n= x)

Discontinued intervention (give reasons) (n=x) Immediately, 30days and 90 days after interventions

Allocated to control (n= 20)

Analysed (n= x)
♦ Excluded from analysis (give reasons) (n= x)

Randomize (n= 40)
