# Supplementary material for: Nurse-led telerehabilitation intervention to improve stroke efficacy: Protocol for a pilot randomized feasibility trial
Source: PLoS One. 2023 Jun 2;18(6):e0280973. doi: 10.1371/journal.pone.0280973 (PMC10237469; doi:10.1371/journal.pone.0280973)
Supplement: Attachment — Submitted filename: Response to Reviewers.docx [file pone.0280973.s005.docx]

Stephanopoulos K.J. Osei,

University of Ghana School of Nursing & Midwifery,

Accra, Ghana.

8^th^ May, 2023.

Doris YP Leung,
Academic Editor,
PLOS ONE.

**Round 2 Response to Editor & Reviewers’ Comments: PONE-D-23-00839**

**Nurse-Led Telerehabilitation Intervention to Improve Stroke Efficacy: Protocol for a pilot randomized feasibility trial**

Dear Dr Leung,

Many thanks for the review once again. In this letter we have included a revised draft of our manuscript Nurse-Led Telerehabilitation Intervention to Improve Stroke Efficacy: Protocol for a pilot randomized feasibility trial” to PLOS ONE addressing the comments of the reviewers and the journal. We appreciate the time and effort that you and the reviewers have dedicated to providing brilliant feedback on the manuscript.

| **Additional Editor’s comments** | **Response** |
| --- | --- |
| 1. Line 312: Will the data collection at baseline be done before intervention or before randomization? | Thank you for question.  The correction has been made in line 285. Essentially, baseline data would be gathered on 2 occasions and all two occasions have been clearly defined |
| 2. Line 322: SKJO will not be blinded of group allocation as SKJO will open the envelopes for randomization (line 199) and hence should not be involved in data collection after group allocation. In addition, some typos were found | Many thanks for pointing this out.  SKJO and other investigators have been removed as assessors of patient outcomes in line 298 and 184. Instead, independent assessors who are blind to group allocation would be employed for this role. |
| 3. Line 191: Typo “She will use of combination” | Typo rectified in line 171 |
| 4. Line 195: ‘researcher’ instead of ‘research’ | Type rectified in line 175 |

| **Journal requirement** | **Response** |
| --- | --- |
| Please review your reference list to ensure that it is complete and correct. If you have cited papers that have been retracted, please include the rationale for doing so in the manuscript text, or remove these references and replace them with relevant current references. Any changes to the reference list should be mentioned in the rebuttal letter that accompanies your revised manuscript. If you need to cite a retracted article, indicate the article’s retracted status in the References list and also include a citation and full reference for the retraction notice. | The following references were added to the manuscript.  25. Meng X, Chen X, Liu Z, Zhou L. Nursing practice in stroke rehabilitation: Perspectives from multi‐disciplinary healthcare professionals. Nursing & Health Sciences. 2019 Aug 15;22(1):28–37.  26. Lenz ER, Shortridge-Baggett LM. Self-Efficacy In Nursing: Research and Measurement Perspectives. Google Books. Springer Publishing Company; 2002.  27. Bandura A. Self-efficacy: The exercise of control. New York: Freeman; 1997.  28. Korpershoek C, van der Bijl J, Hafsteinsdóttir TB. Self-efficacy and its influence on recovery of patients with stroke: a systematic review. Journal of Advanced Nursing. 2011 Jun 7;67(9):1876–94.  None of the initial references were retracted from the study |

Thank you for considering our manuscript.

Sincerely,

Stephanopoulos Osei (BSN, RN).
